# Prediction of treatment dosage and duration from free-text prescriptions: an application to ADHD medications in the Swedish prescribed drug register

**DOI:** 10.1136/ebmental-2020-300231

**Published:** 2021-04-01

**Authors:** Le Zhang, Tyra Lagerberg, Qi Chen, Laura Ghirardi, Brian M D'Onofrio, Henrik Larsson, Alexander Viktorin, Zheng Chang

**Affiliations:** 1 Department of Medical Epidemiology and Biostatistics, Karolinska Institute, Stockholm, Sweden; 2 Department of Psychological and Brain Sciences, Indiana University, Bloomington, Indiana, USA; 3 School of Medical Sciences, Örebro Universitet, Örebro, Sweden

## Abstract

**Background:**

Accurate estimation of daily dosage and duration of medication use is essential to pharmacoepidemiological studies using electronic healthcare databases. However, such information is not directly available in many prescription databases, including the Swedish Prescribed Drug Register.

**Objective:**

To develop and validate an algorithm for predicting prescribed daily dosage and treatment duration from free-text prescriptions, and apply the algorithm to ADHD medication prescriptions.

**Methods:**

We developed an algorithm to predict daily dosage from free-text prescriptions using 8000 ADHD medication prescriptions as the training sample, and estimated treatment periods while taking into account several features including titration, stockpiling and non-perfect adherence. The algorithm was implemented to all ADHD medication prescriptions from the Swedish Prescribed Drug Register in 2013. A validation sample of 1000 ADHD medication prescriptions, independent of the training sample, was used to assess the accuracy for predicted daily dosage.

**Findings:**

In the validation sample, the overall accuracy for predicting daily dosage was 96.8%. Specifically, the natural language processing model (NLP1 and NLP2) have an accuracy of 99.2% and 96.3%, respectively. In an application to ADHD medication prescriptions in 2013, young adult ADHD medication users had the highest probability of discontinuing treatments as compared with other age groups. The daily dose of methylphenidate use increased with age substantially.

**Conclusions:**

The algorithm provides a flexible approach to estimate prescribed daily dosage and treatment duration from free-text prescriptions using register data. The algorithm showed a good performance for predicting daily dosage in external validation.

**Clinical implications:**

The structured output of the algorithm could serve as basis for future pharmacoepidemiological studies evaluating utilization, effectiveness, and safety of medication use, which would facilitate evidence-based treatment decision-making.

## Background

Electronic healthcare databases have become widely available in the past two decades,^1 2^ and provide a valuable source for generating real-world evidence on utilisation, effectiveness and safety of pharmacological treatments.^3 4^ Pharmacoepidemiological studies using electronic healthcare databases rely on accurate measurements of treatment dosage and duration. However, the quality of these measurements varies by database and region.^2^ Some databases (e.g. the Ontario Drug Benefit database)^5^ contain structured information on treatment dosage and duration (e.g. days of supply), while others do not. For the latter, special methods are needed to address the limitations.

The Swedish Prescribed Drug Register,^6^ similar to some other databases in Europe (e.g. the Danish National Prescription Registry),^7^ contains detailed information on the type of prescribed medicine, dispensation date and amount of dispensed medication, but contains no structured variable for daily dosage or duration. Instead, daily dosage information is provided in an unstructured ‘free-text’ variable. Two types of methods have been proposed to estimate treatment dosage and duration in such settings. A first type of method estimates treatment periods based on the sequence of dispensed medications. A simple version of this method defines any prescriptions falling within a time interval of each other to belong to a continuous treatment period.^8 9^ A more advanced approach uses the defined daily doses of the purchased medication over time to estimate continuous treatment periods.^10^ This type of method relies on information from future prescriptions when determining the treatment status at a specified time point. This may induce bias when prescribing or purchasing behaviours are influenced by the outcome of interest (e.g. substance abuse events could influence subsequent prescription of medications used to treat attention-deficit/hyperactivity disorder (ADHD)).^11^ A second type of method first extracts prescribed daily dosage by applying a set of syntax rules to free-text prescription (‘text parsing’),^12–14^ and then estimates treatment duration based on the amount of dispensed medication and estimated daily dosage. This method avoids bias that arises from relying on future information. However, the syntax rules are usually not flexible enough to cover all possible ways that daily dosage is described in free-text. In addition, previous studies using text-parsing methods have not accounted for prescriptions where the free text was missing or non-informative.^12–14^ Another important limitation of existing methods is that they do not fully account for titrated prescriptions (i.e. stepwise changes in dosage over treatment time), which may affect estimates of both prescribed daily dosage and treatment duration. Given the limitations of existing methods, a flexible approach that does not rely on future information, and accounts for both missing prescription information and titrated dosages, is desirable.

The need for a flexible and accurate approach for estimating prescribed dosage and treatment duration is highly relevant to ADHD medications. Medications for ADHD are increasingly used in many countries^15^ and there is a growing number of pharmacoepidemiological studies on the utilisation, effectiveness and safety of ADHD medications based on electronic healthcare databases.^3^ There are, however, several challenges to accurately identify the dosage and duration of ADHD treatment using prescription data. First, therapeutic levels of medications are typically achieved through dosage titration—usually, a low initial dosage is increased over several weeks.^16^ Second, discontinuation of pharmacological treatment for ADHD is common due to various reasons.^17 18^ Finally, there is considerable between-individual variability in the pharmacokinetics of ADHD medications, resulting in large variation in prescribed daily dosage.^19^ A method that can accurately capture all these aspects of ADHD medication treatment from electronic healthcare databases is of high priority to advance the understanding of risks and benefits of ADHD medication use.

## Objective

In this study, we aimed to (1) develop and evaluate a new algorithm that employs machine learning to predict prescribed daily dosage from free-text prescription and uses the predictions to estimate continuous treatment periods; (2) examine the patterns of ADHD medication treatment by applying the algorithm to dispensed prescriptions of ADHD medications from the Swedish Prescribed Drug Register.

## Methods

### Data source

The Swedish Prescribed Drug Register contains information on all prescribed pharmaceuticals dispensed at pharmacies nationwide since July 2005.^6^ Collected information includes unique personal identification numbers, drug identity, strength of the drug, package size, number of packages dispensed, Nordic article number, dates of dispensation from a pharmacy, the prescriber’s specialisation, costs and a free-text variable that includes treatment instructions from the prescriber. The drug identity is coded using the Anatomical Therapeutic Chemical (ATC) classification system. The trade name, dosage strength, pharmaceutical form, package size and type of package related to each dispensed prescription is identifiable by the Nordic article number, applied to identify medicines with marketing authorisation in the Nordic countries.^20^ In the unstructured free-text prescription variable, the prescriber usually indicates the amount of medication the patient should take (mostly by specifying quantity, frequency and dose form), and whether dosage should change over time (titration).

### Algorithm development

**Figure 1:**
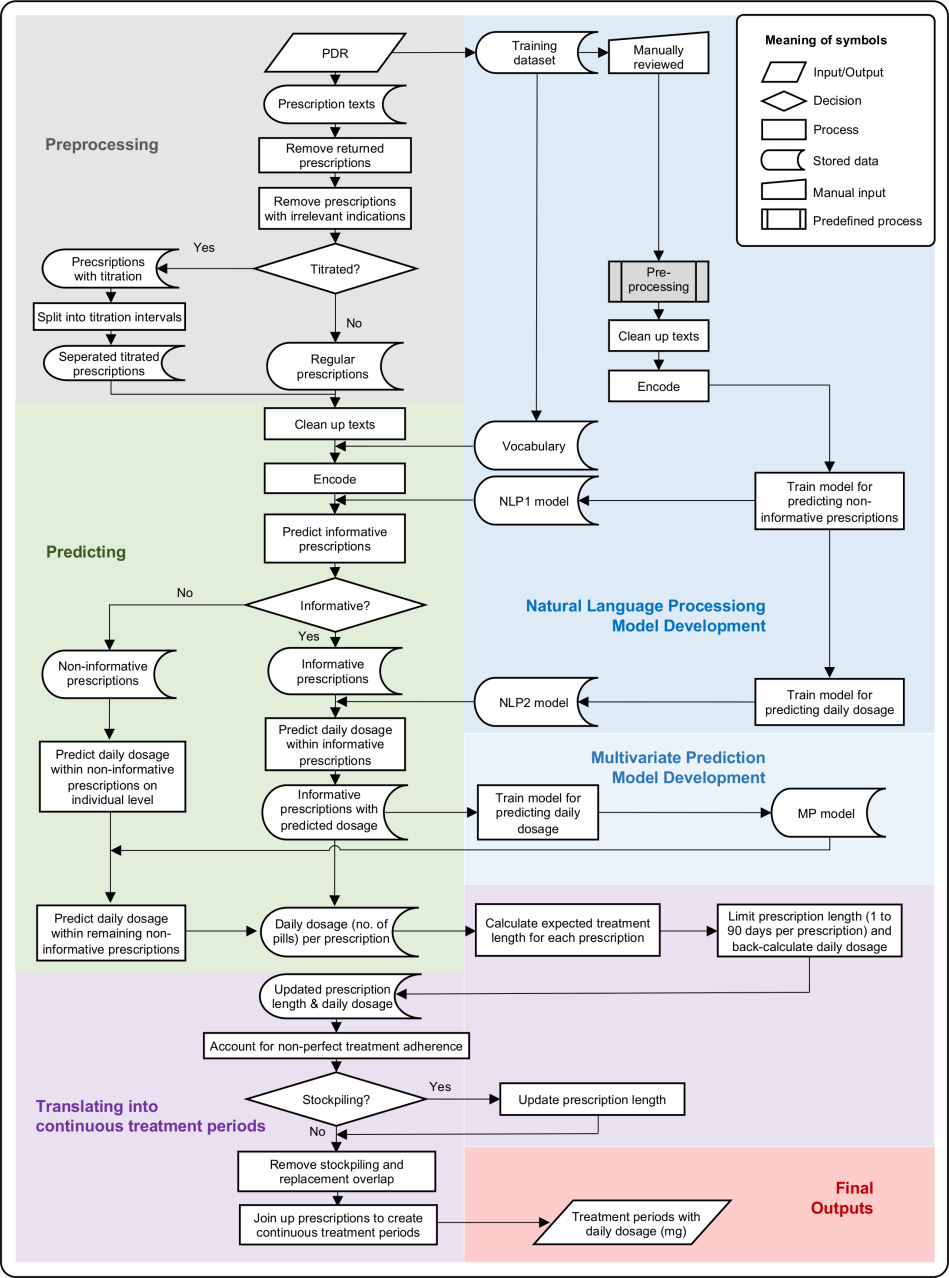
Workflow of prediction of prescribed daily dosage from individual prescriptions and estimation of continuous treatment periods. PDR, Prescribed Drug Register; NLP, natural language processing model.

The process workflow from prescription texts to prediction of dosage and estimation of continuous treatment periods is presented in figure 1.

#### Preprocessing

The prescription data first went through a preprocessing step (figure 1). First, we removed prescriptions that were returned to pharmacies by patients after dispensation. Second, we excluded prescriptions for indications other than ADHD (e.g. narcolepsy, multiple sclerosis, see online supplemental table S1 for a complete list of keywords). Third, we identified titrated prescriptions and split them so that each titration interval was processed as a separate entry in the natural language processing models (NLP) (see step 2). This step was achieved by identifying a set of keywords representing titration and then splitting up prescriptions based on syntax rules. Finally, all prescriptions (titration-split and regular) were entered into a text cleaning procedure. Non-informative punctuation was removed, Swedish stop words were filtered away, each word was stemmed to its base form without grammatical inflections (e.g. ‘tabletten’ became ‘tablet’), each prescription was tokenised so that each word was represented by a unique number, and then padded to ensure the same sequence length of each prescription.^21^

10.1136/ebmental-2020-300231.supp1 Supplementary data

#### NLP model development

##### Training sample

After preprocessing, we extracted 8000 ADHD medication prescriptions from the Swedish Prescribed Drug Register 2006–2013 (6500 prescriptions for methylphenidate (ATC code: N06BA04), and 500 prescriptions each for atomoxetine (N06BA01), amfetamine (N06BA01) and dexamfetamine (N06BA02)) as the training dataset, titration-split to 8582 entries (7456 regular and 544 titrated texts). These prescription texts were reviewed by two independent researchers to identify whether the prescription is non-informative (inadequate information to determine daily dosage) and the gold-standard daily dosage (the number of pills prescribed per day). In the case of discrepancy between the records of the two first researchers, a third researcher resolved the difference. When a prescription specified a dosage range or indicated add-on pills, we considered the minimum dosage that an individual took per day. After preprocessing, the 8000 prescription texts were used as a corpus for building a vocabulary, which is a list of words that occurred in prescription texts, each represented by a unique integer value.

##### NLP1 model (identifying non-informative prescription texts)

The first NLP (NLP1) was built to identify whether the prescription texts contained enough information for prediction of daily dosage (‘informative’). The outcome was binary—informative or non-informative prescription text. The NLP1 is a densely connected neural network with back-propagation learning technique to adjust the weights of each connection and reduce the value of the error function.^22^ The training dataset for NLP1 included all titration-split prescription texts, which were first passed into an embedding layer where words were represented in vector space according to their syntactic and semantic inter-relationships,^23^ followed by a flattening layer, a hidden layer and an output layer. The output layer of NLP1 consisted of one node where the probability of being a non-informative prescription was calculated.

##### NLP2 model (predicting daily dosage)

The second NLP (NLP2) was built to predict daily dosage if the prescription text was predicted as informative. The NLP2 model had similar architecture as the NLP1, which also contained an embedding layer, a flattening layer, a hidden layer and an output layer. The output layer consisted of 16 nodes which represented different daily dosage classes, that is, 0.5, 1, 2, 3, 4, 5, 6, 7, 8, 9, 10–11, 12–13, 14–15, 16–17, 18–19 and 20+ pills per day (daily dosage above 10 pills were collapsed due to their low frequencies), with the probabilities of classification into each dosage category.

#### Dosage prediction

##### Dosage prediction among informative prescription texts

After preprocessing, all ADHD medication prescriptions from the Swedish Prescribed Drug Register were fed into the NLP1 model. Prescription texts with a predicted probability higher than 0.5 in this model were defined as non-informative prescriptions, and the rest (informative) prescriptions were passed into the NPL2 model to predict daily dose. The dosage with the highest predicted probability was chosen as the predicted daily dosage. Any prescription texts with a maximum predicted probability lower than 0.5 in this step were also labelled as non-informative and passed into the next step.

##### Dosage prediction among non-informative prescription texts

All prescriptions predicted as non-informative were processed in this step. The non-informative prescriptions were first predicted with individual prescription record. Estimated prescribed daily dosage within 1 year prior the non-informative prescription of the same medication was assumed to be the current daily dosage for each individual, on the assumption that the prescribing clinician may not restate the dose if the patient already has it from an earlier prescription of the same medication. The remaining non-informative prescription texts were then predicted using a multivariate random forest prediction model. Prescription texts with predicted probability higher than 0.5 in step 3 were used as training data in this step. Based on the information available in the Swedish Prescribed Drug Register, we identified six relevant predictors, including age, sex, specialty of prescriber (categorised into 6 classes), county of prescription (categorised into 20 classes), Nordic article number, and sequence of prescription within a 6-month treatment period. Each random forest model comprised 100 individual base learners where each base learner was trained using a subset of random samples from the training data. Results of dosage prediction were generated by returning the majority of the dosage class predicted by all individual trees to reduce overfitting and increase generalisability.^24^

#### Validation of the algorithm for predicting daily dosage

To assess the performance of the algorithm, we randomly selected 1000 prescriptions of ADHD medications in 2013 from the Swedish Prescribed Drug Register (independent from the training sample). Gold-standard values were reviewed the same way as for training data. Accuracies of non-informative prescription prediction and dosage prediction were calculated by comparing the predicted values and gold standard values. We calculated 95% CIs for the accuracy of NLP1 and NLP2 using the Wilson Score Interval. We also calculated the weighted g-means metric for both models, in order to account for the imbalanced distribution of the different class labels.^25^ In addition to these measures, we reported the overall accuracy of the two models (i.e. one minus the percentage of erroneous predictions among those prescriptions classified as informative).

#### Estimation of continuous treatment periods

Continuous treatment periods were generated in several steps (figure 1). First, expected treatment length of each dispensation was calculated by dividing the number of pills in the package(s) by the predicted daily dosage (i.e. number of pills, see online supplemental text for titrated prescriptions). Second, the minimum expected treatment length for each dispensation was set at 1 day, and the maximum at 90 days (a prescribed medication should be dispensed for at most 3 months at a time to be reimbursed).^26^ If the expected treatment length exceeded this range, the daily dosage was updated with the number of pills in the package(s) divided by the new expected prescription length. Third, the expected treatment lengths were extended by a coefficient of non-perfect adherence. The coefficient here was considered to be 7/5, assuming ADHD medications to be consumed on weekdays and sometimes omitted on weekends. Fourth, stockpiling was taken into account. Stockpiling is a phenomenon where patients collect prescription refills from the pharmacy before their current prescription has run out. If a stockpiling instance was identified (a description of the stockpiling conditions can be found in online supplemental text), the stockpiled prescription and current prescription were joined up into a continuous treatment period. Fifth, dosage in weight form associated with each treatment period was calculated using the strength (amount of active substance) of the medication, indicated by the package information for the given Nordic article number. Sixth, for each medication type (by ATC code), if the treatment length of a dispensed prescription reaches the next dispensation, they will be considered as a continuous treatment period. Overlapping prescriptions were assumed to be consumed simultaneously unless they were the same drug. The final output of continuous treatment period was stratified by changes in the dosage (in weight form).

All data management was performed using SAS V.9.4 (SAS Institute). The core processes of the algorithm were carried out in Python (V.3.7.1) using Keras.

### Patterns of ADHD medication use

#### Treatment discontinuation of ADHD medications

To examine treatment discontinuation pattern of ADHD medications, we identified incident ADHD medication users aged 6 or above in 2013. A 1-year wash-out period was applied to ascertain incident medication use. Medication users were followed from the date of incident ADHD medication dispensation until the date when treatment periods discontinued or 31 December 2013. Kaplan-Meier analysis was used to estimate the proportions of treatment discontinuation stratified by sex and age groups (children (6–12 years), adolescents (13–17 years), young adults (18–29 years), middle-aged adults (30–49 years) and older adults (above 50 years)). A sensitivity analysis was performed in incident ADHD medication users excluding those with a single ADHD medication prescription.

#### Prescribed daily dose (in weight form) of methylphenidate

We examined the average prescribed daily dose (mg/day) of methylphenidate for all methylphenidate users aged 6 or above in 2013. If an individual had more than one treatment period, dose of the longest treatment period was considered. The prescribed daily dose was grouped into three categories: low (0–30 mg), medium (31–60 mg) and high (above 60 mg),^27^ and presented by sex and age groups.

## Findings

After implementing the algorithm, predicted daily dosage for all ADHD medication prescriptions in 2013 (647 519 prescriptions) were generated. Distribution of predicted daily dosage for each type of ADHD medication is shown in online supplemental table S2.

### Validation of the algorithm for predicting daily dosage

Among the validation dataset of 1000 prescriptions, there were 853 methylphenidate, 118 atomoxetine, 7 amphetamine and 22 dexamphetamine prescriptions. For all prescriptions (regular and titrated prescriptions), the accuracy in predicting non-informative prescriptions (NLP1 model) was 99.2% (95% CI 98.6% to 99.8%), and the accuracy for dosage prediction (NLP2 model) was 96.3% (95% CI 95.1% to 97.6%) among those prescriptions with defined gold-standard dosage (table 1). The G-means statistic across ADHD medication types was 0.973 (95% CI 0.963 to 0.983) for NLP1, and 0.968 (95% CI 0.956 to 0.980) for NLP2. The overall accuracy of the algorithm for predicting daily dosage was 96.8% (95% CI 95.7% to 97.9%) with 96.8% (95% CI 95.7% to 97.9%) for regular prescriptions and 94.4% (95% CI 83.9% to 100%) for titrated prescriptions. The overall accuracy was 96.5% (95% CI 95.2% to 97.7%) for methylphenidate, 100.0% for atomoxetine, 85.7% (95% CI 59.8% to 100%) for amphetamine and 95.5% (95% CI 86.7% to 100%) for dexamphetamine (online supplemental table S3). The performance of the dosage prediction model in different daily dosage classes is shown in online supplemental figure S1 and the confusion matrix is shown in online supplemental figure S2. An overall good performance among ADHD medication prescriptions was indicated comparing the gold standard daily dosage to the predicted daily dosage. Finally, online supplemental table S4 gives the number of errors by error size (≤20%, 20%–50% and >50%) and dosage class. The distribution of dosage classes (gold standard) in training versus validation sample is shown in online supplemental table S5.

**Table 1:** Performance of the algorithm for predicting daily dosage in the validation sample

Models	Prescription type	N	Accuracy (95% CI)	G-means (95% CI)
Non-informativeness prediction (NLP1 model)	Regular prescriptions	982	0.992 (0.986 to 0.998)	0.969 (0.958 to 0.980)
	Titrated prescriptions	18	1.000 (1.000 to 1.000)	1.000 (1.000 to 1.000)
	All prescriptions	1000	0.992 (0.968 to 0.998)	0.973 (0.963 to 0.983)
Dosage prediction (NLP2 model)	Regular prescriptions	852	0.964 (0.951 to 0.976)	0.969 (0.957 to 0.981)
	Titrated prescriptions	18	0.944 (0.839 to 1.000)	0.972 (0.896 to 1.000)
	All prescriptions	870	0.963 (0.951 to 0.976)	0.968 (0.956 to 0.980)
Overall prediction	Regular prescriptions	982	0.968 (0.957 to 0.979)	N/A
	Titrated prescriptions	18	0.944 (0.839 to 1.000)	N/A
	All prescriptions	1000	0.968 (0.957 to 0.979)	N/A

N/A, not available; NLP1, first natural language processing model; NLP2, second natural language processing model.

### Patterns of ADHD medication use

#### Treatment discontinuation of ADHD medications

**Figure 2:**
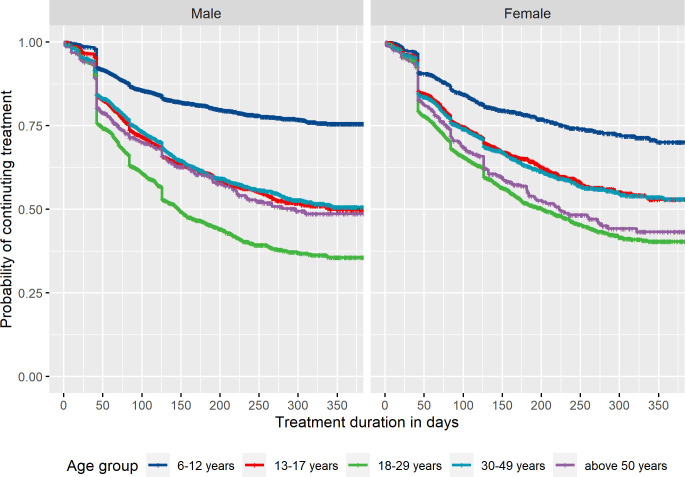
Kaplan-Meier plot of time to treatment discontinuation among incident ADHD medication users in 2013, stratified by sex.

We identified 21 745 incident ADHD medication users aged 6 or above in 2013. Young adults had the highest probability of treatment discontinuation, with only 35.6% (95% CI 33.0% to 38.4%) of males and 40.4% (95% CI 37.6% to 43.3%) of females estimated to remain on treatment after 1 year (figure 2). Children had the lowest treatment discontinuation rate, with 75.6% (95% CI 73.7% to 77.5%) of males and 70.0% (95% CI 66.1% to 74.2%) of females estimated to remain on treatment after 1 year. In males, adolescents, middle-aged adults and older adults had similar treatment discontinuation pattern, with 48.6%–50.6% estimated to remain on treatment after 1 year. In females, the proportion were lower among older adults (43.3%) than among adolescents (52.9%) and middle-aged adults (53.1%). Similar patterns of treatment discontinuation across age groups were found after excluding those with a single dispensed prescription (online supplemental figure S3).

#### Prescribed daily dose of methylphenidate

**Figure 3:**
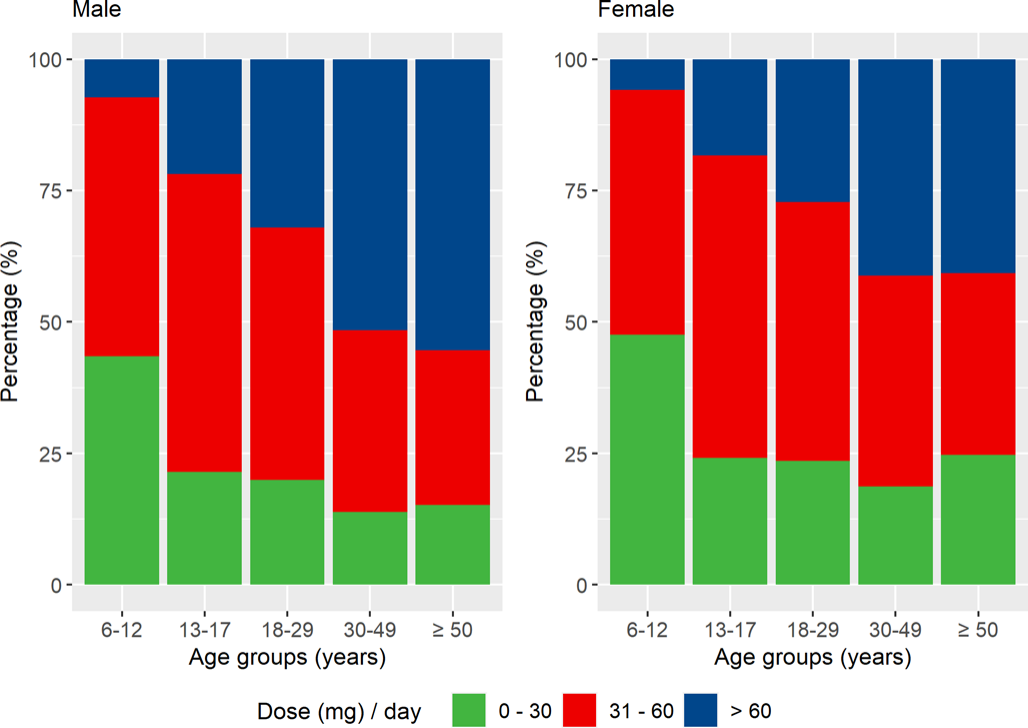
Prescribed daily dose (in weight form) of methylphenidate across age groups, stratified by sex.

A total of 66 187 individuals aged 6 or above received methylphenidate in 2013. The prescribed daily dose increased substantially with age in both males and females (figure 3). In males, 7.3% of children had a high dose (>60 mg/day), while the percentage increased from 21.9% in adolescents to 55.4% in older adults. The percentage of females receiving a high dose also increased with age, from 5.8% in children to 40.7% in older adults.

## Discussion

We developed an algorithm that predicts prescribed daily dosage from free-text prescriptions, and uses these predictions to estimate treatment duration, taking into account important features such as dosage titration and missing dosage texts. The algorithm showed a good performance for predicting daily dosage, with an overall accuracy of 96.8% in the validation sample. When our algorithm was applied to ADHD prescriptions to estimate treatment duration based on the predicted daily dosage, we found that the rate of discontinuing treatment varied substantially between age groups and that the prescribed daily dose of methylphenidate increased with age.

Before considering the use of a prediction models, external validation is essential (i.e. evaluating the performance of the model in a sample that was not used to develop the model). Our model for dosage prediction was 96.8% accurate in the validation sample. This compares favourably to other algorithms that have been evaluated externally—for example, Shah *et al* (93.0% accuracy)^14^ and Karystianis *et al* (90.9% accuracy).^13^ Unfortunately, the performance of many other existing algorithms has only been assessed internally—for example, McTaggart *et al* (94.2% accuracy),^12^ and Morin (96% accuracy).^28^

The algorithm provided a structured output, including treatment duration, start date of treatment, end date of treatment and prescribed daily dosage (in weight form) during treatment, which could be used for pharmacoepidemiological studies on drug utilisation, effectiveness and safety. Our method for joining up individual prescriptions into continuous periods takes stockpiling and non-perfect adherence into account, since circumstances such as lack of treatment adherence and deviations from the treatment plan may occur.^11^ Application of our algorithm to ADHD medication prescriptions provided clinically plausible patterns of medication use across age groups. In line with previous research,^18^ we found that discontinuation of ADHD medication varied with age, with highest discontinuation rate among young adults. Discontinuation of ADHD medication could be due to adverse effects and/or lack of treatment effectiveness.^17 18^ Meanwhile, some individuals remit from ADHD (around 35% by the age of 25 years),^29^ meaning they do not seek further pharmacological treatment. Second, we found substantial variation in the prescribed daily dose of methylphenidate between individuals and across age groups, highlighting the importance of an individualised dose-optimisation treatment approach—consistent with guidelines for ADHD treatment.^30^

The method for dosage prediction in our algorithm relied on only a few assumptions, making it more flexible than previous methods. Future extensions of our algorithm could provide more tunable parameters in the dosage prediction step, covering features that are likely to vary by medication type, geographical region and prescription practices. Our method for treatment duration estimation makes several assumptions based on our target medication and Swedish setting. Different assumptions may be relevant to reflect prescription practices for other medications and/or settings.

Our algorithm has several strengths. First, we use machine-learning to process the free-text prescriptions without relying on prespecified sets of rules, which allows for greater flexibility and easier adaptation of our algorithm to other linguistic settings. Second, the method takes titration and missing daily dosage information into account. Finally, it relies minimally on future prescription information when estimating the duration of treatment from a given prescription, which is expected to generate less biased estimates of treatment duration. Our algorithm also has some limitations. The performance appeared to be lower in prescriptions that have complex instructions, which is often associated with high number of daily dosages. This was indicated by the fact that the predictive performance was better for atomoxetine prescriptions, which had a smaller variation in prescribed daily dosages, than for methylphenidate prescriptions. Another limitation is that the prediction of daily dosage and treatment duration was based on dispensed prescriptions, but we were unable to verify whether the dispensed medication was consumed. Further developments could be made to make our algorithm more flexible, for example, providing dosage predictions of different probabilities, predicting a range in dosage quantity/frequency (maximum, minimum or ‘as needed’) and/or allowing more flexible assumptions when estimating continuous treatment periods for different types of medication.

In conclusion, we have developed an algorithm to predict prescribed daily dosage and estimate treatment duration from free-text prescriptions. Our algorithm performs well for dosage prediction in external validation, and the estimated dosage and duration of ADHD medication use correspond well to expected usage patterns across age groups. The structured output of the algorithm could serve as basis for future pharmacoepidemiological studies evaluating utilisation, effectiveness and safety of medication use.

## Data Availability

Data may be obtained from a third party and are not publicly available. The electronic health records record patient identifiable information and therefore cannot be shared publicly. The data can be used and reused by applying via Socialstyrelsen (https://bestalladata.socialstyrelsen.se/)

## References

[1] Jensen PB , Jensen LJ , Brunak S . Mining electronic health records: towards better research applications and clinical care. Nat Rev Genet 2012;13:395–405. 10.1038/nrg3208 22549152

[2] Murray ML , Insuk S , Banaschewski T , et al . An inventory of European data sources for the long-term safety evaluation of methylphenidate. Eur Child Adolesc Psychiatry 2013;22:605–18. 10.1007/s00787-013-0386-x 23508655 PMC3830128

[3] Chang Z , Ghirardi L , Quinn PD , et al . Risks and benefits of attention-deficit/hyperactivity disorder medication on behavioral and neuropsychiatric outcomes: a qualitative review of pharmacoepidemiology studies using linked prescription databases. Biol Psychiatry 2019;86:335–43. 10.1016/j.biopsych.2019.04.009 31155139 PMC6697582

[4] Davis KAS , Farooq S , Hayes JF , et al . Pharmacoepidemiology research: delivering evidence about drug safety and effectiveness in mental health. Lancet Psychiatry 2020;7:363-370. 10.1016/S2215-0366(19)30298-6 31780306

[5] Burden AM , Paterson JM , Gruneir A , et al . Adherence to osteoporosis pharmacotherapy is underestimated using days supply values in electronic pharmacy claims data. Pharmacoepidemiol Drug Saf 2015;24:67–74. 10.1002/pds.3718 25331490

[6] Wettermark B , Hammar N , Fored CM , MichaelFored C , et al . The new Swedish Prescribed Drug Register--opportunities for pharmacoepidemiological research and experience from the first six months. Pharmacoepidemiol Drug Saf 2007;16:726–35. 10.1002/pds.1294 16897791

[7] Kildemoes HW , Sørensen HT , Hallas J . The Danish national prescription registry. Scand J Public Health 2011;39:38–41. 10.1177/1403494810394717 21775349

[8] Lichtenstein P , Halldner L , Zetterqvist J , et al . Medication for attention deficit–hyperactivity disorder and criminality. N Engl J Med Overseas Ed 2012;367:2006–14. 10.1056/NEJMoa1203241 PMC3664186 23171097

[9] Chang Z , Lichtenstein P , Långström N , et al . Association between prescription of major psychotropic medications and violent Reoffending after prison release. JAMA 2016;316:1798–807. 10.1001/jama.2016.15380 27802545 PMC5100822

[10] Tanskanen A , Taipale H , Koponen M , et al . From prescription drug purchases to drug use periods – a second generation method (PRE2DUP). BMC Med Inform Decis Mak 2015;15:21. 10.1186/s12911-015-0140-z 25890003 PMC4382934

[11] Nielsen LH , Løkkegaard E , Andreasen AH , et al . Using prescription registries to define continuous drug use: how to fill gaps between prescriptions. Pharmacoepidemiol Drug Saf 2008;17:384–8. 10.1002/pds.1549 18213736

[12] McTaggart S , Nangle C , Caldwell J , et al . Use of text-mining methods to improve efficiency in the calculation of drug exposure to support pharmacoepidemiology studies. Int J Epidemiol 2018;47:617–24. 10.1093/ije/dyx264 29420741 PMC5913611

[13] Karystianis G , Sheppard T , Dixon WG , et al . Modelling and extraction of variability in free-text medication prescriptions from an anonymised primary care electronic medical record research database. BMC Med Inform Decis Mak 2016;16:18. 10.1186/s12911-016-0255-x 26860263 PMC4748480

[14] Shah AD , Martinez C . An algorithm to derive a numerical daily dose from unstructured text dosage instructions. Pharmacoepidemiol Drug Saf 2006;15:161–6. 10.1002/pds.1151 16170830

[15] Raman SR , Man KKC , Bahmanyar S , et al . Trends in attention-deficit hyperactivity disorder medication use: a retrospective observational study using population-based databases. Lancet Psychiatry 2018;5:824–35. 10.1016/S2215-0366(18)30293-1 30220514

[16] Greenhill LL , Swanson JM , Vitiello B , et al . Impairment and deportment responses to different methylphenidate doses in children with ADHD: the MTA titration trial. J Am Acad Child Adolesc Psychiatry 2001;40:180–7. 10.1097/00004583-200102000-00012 11211366

[17] Charach A , Fernandez R . Enhancing ADHD medication adherence: challenges and opportunities. Curr Psychiatry Rep 2013;15:371. 10.1007/s11920-013-0371-6 23712722 PMC3718998

[18] Zetterqvist J , Asherson P , Halldner L , et al . Stimulant and non-stimulant attention deficit/hyperactivity disorder drug use: total population study of trends and discontinuation patterns 2006-2009. Acta Psychiatr Scand 2013;128:70–7. 10.1111/acps.12004 22943458

[19] Frölich J , Banaschewski T , Döpfner M , et al . An evaluation of the pharmacokinetics of methylphenidate for the treatment of attention-deficit/ hyperactivity disorder. Expert Opin Drug Metab Toxicol 2014;10:1169–83. 10.1517/17425255.2014.922542 24856438

[20] The Nordic number centre. Nordic article number (Vnr): instruction booklet, 2019. Available: http://wiki.vnr.fi/wp-content/uploads/2019/02/Nordic-Article-Numbers-Nordic-instructions-ver-2.0.pdf [Accessed 22 Jul 2019].

[21] Kannan S , Gurusamy V . Preprocessing techniques for text mining. International Journal of Computer Science & Communication Networks 2014;5:7–16.

[22] Hecht-Nielsen R . Theory of the backpropagation neural network. Neural networks for perception: Elsevier, 1992: 65–93.

[23] Mikolov T , Chen K , Corrado G . Efficient estimation of word representations in vector space. arXiv 2013.

[24] Criminisi A , Shotton J , Konukoglu E . Decision forests: a unified framework for classification, regression, density estimation, manifold learning and Semi-Supervised learning. Foundations and Trends® in Computer Graphics and Vision 2011;7:81–227. 10.1561/0600000035

[25] Fernández A , García S , Galar M . Learning from imbalanced data sets. Springer, 2018.

[26] Fazel S , Zetterqvist J , Larsson H , et al . Antipsychotics, mood stabilisers, and risk of violent crime. Lancet 2014;384:1206–14. 10.1016/S0140-6736(14)60379-2 24816046 PMC4165625

[27] Huss M , Duhan P , Gandhi P , et al . Methylphenidate dose optimization for ADHD treatment: review of safety, efficacy, and clinical necessity. Neuropsychiatr Dis Treat 2017;13:13. 10.2147/NDT.S130444 PMC5505611 28740389

[28] Morin L . Too much, too late? drug prescribing for older people near the end of life, 2019.

[29] Faraone SV , Biederman J , Mick E . The age-dependent decline of attention deficit hyperactivity disorder: a meta-analysis of follow-up studies. Psychol Med 2006;36:159–65. 10.1017/S003329170500471X 16420712

[30] Swedish national board of health and welfare (Swedish: Socialstyrelsen). drug treatment of ADHD in children and adults: support for treatment decisions, 2014. Available: https://www.socialstyrelsen.se/globalassets/sharepoint-dokument/artikelkatalog/ovrigt/2015-4-14.pdf

