# Supplementary material for: Prediction of treatment dosage and duration from free-text prescriptions: an application to ADHD medications in the Swedish prescribed drug register
Source: Evid Based Ment Health. 2021 Apr 1;24(4):146–52. doi: 10.1136/ebmental-2020-300231 (PMC8543229; doi:10.1136/ebmental-2020-300231)

Supplements

Supplementary Text

Estimation of treatment length in titrated prescriptions

If a prescription was titrated, the expected treatment length for each titration period was extracted from the text, if available. If the length of the last titration period was not specified in the text, the remaining number of pills in the last titration period was calculated by subtracting the sum of the daily dosage multiplied by titration length in preceding titration periods from the total number of pills in the package(s) of the prescription. This number was then divided by the daily dosage specified in the last titration period to obtain the titration period treatment length. Missing values of titration length in other titration periods were set to be 7 days, based on a review of titrated prescriptions in the training dataset.

Identification of stockpiling

We define any prescription as an instance of stockpiling where the current (i), previous (i-1) and next (i+1) prescription fulfill the following conditions apply:

a) The current (i), previous (i-1) and next (i+1) prescription have the same article number and predicted daily dosage, AND
b) There is an overlap between the current (i) and previous (i-1) prescriptions, AND
c) There is non-overlap (a gap) between the current (i) and next (i+1) prescriptions, AND
d) The gap is smaller than 90 days when not adjusted for non-perfect adherence (i.e. 126 days when adjusted for non-perfect adherence in our example), AND
e) The end of the previous prescription (i-1) length does not overlap with the start of the next (i+1), AND
f) The end of the previous prescription (i-1) does not exceed the end of the current prescription (i).

If stockpiling applied, the overlap between the current (i) and previous (i-1) prescription was added to the length of the current prescription. If this overlap exceeded the length of the gap between the current (i) and next (i+1) prescription, these prescriptions were joined up into a continuous treatment period.

Table S1. List of indications of off-label use of ADHD medications.

Swedish	English
ms	multiple sclerosis
multipel skleros	multiple sclerosis
narko	narcolepsy
narco	narcolepsy
tröt	tiredness
fatiq	fatigue
fatig	fatigue
handikappande	disabling
hypersomni	hypersomnia
sömnighet	somnolence
idiopatisk	idiopathic
kataplexi	cataplexy
smärta	pain

Table S2. Distribution of predicted prescribed daily dose, by type of ADHD medication.

No. of pills per day	Methylphenidate	Atomoxetine	Amphetamine	Dexamphetamine
0.5	1447 (0.26%)	97 (0.12%)	33 (0.61%)	99 (0.69%)
1	388958 (69.48%)	61826 (76.22%)	354 (6.51%)	671 (4.67%)
2	96921 (17.31%)	15308 (18.87%)	412 (7.57%)	475 (3.31%)
3	32671 (5.84%)	2563 (3.16%)	574 (10.55%)	755 (5.25%)
4	16916 (3.02%)	622 (0.77%)	592 (10.88%)	858 (5.97%)
5	6393 (1.14%)	212 (0.26%)	300 (5.51%)	942 (6.55%)
6	6280 (1.12%)	129 (0.16%)	494 (9.08%)	1602 (11.15%)
7	3228 (0.58%)	46 (0.06%)	219 (4.02%)	904 (6.29%)
8	1757 (0.31%)	68 (0.08%)	473 (8.69%)	1428 (9.94%)
9	1679 (0.3%)	47 (0.06%)	260 (4.78%)	752 (5.23%)
10-11	718 (0.13%)	70 (0.09%)	173 (3.18%)	1207 (8.4%)
11-12	850 (0.15%)	11 (0.01%)	192 (3.53%)	1019 (7.09%)
14-15	825 (0.15%)	16 (0.02%)	366 (6.73%)	1517 (10.56%)
16-17	559 (0.1%)	22 (0.03%)	216 (3.97%)	922 (6.42%)
18-19	238 (0.04%)	45 (0.06%)	106 (1.95%)	267 (1.86%)
20+	338 (0.06%)	34 (0.04%)	678 (12.46%)	954 (6.64%)
Total	559778 (100%)	81116 (100%)	5442 (100%)	14372 (100%)

Models	Methylphenidate		Atomoxetine		Amphetamine		Dexamphetamine	
	N	Accuracy (%)	N	Accuracy (%)	N	Accuracy (%)	N	Accuracy (%)
Non-informativeness prediction (NLP1 model)	853	99.3 (98.7-99.9)	118	99.2 (97.5-100)	7	100 (100-100)	22	95.5 (86.7-100)
Dosage prediction (NLP2 model)	744	96.0 (94.6-97.4)	104	100 (100-100)	5	80.0 (44.9-100)	17	94.1 (82.9-100)
Overall prediction	853	96.5 (95.2-97.7)	118	100 (100-100)	7	85.7 (59.8-100)	22	95.5 (86.7-100)

Table S3. Accuracy of the algorithm for predicting prescribed daily dosage by medication type.

Table S4. Difference in misclassifications for predicting prescribed daily dosage (NLP2 model) among ADHD medication prescriptions.

Dosage class	N (Gold standard)	N prescriptions with errors	Number of errors by size		
			<=20%	20-50%	>50%
**0.5**	8	5	0	0	5
**1**	601	8	0	1	7
**2**	135	3	0	3	0
**3**	48	4	0	2	2
**4**	19	1	0	1	0
**5**	9	2	2	0	0
**6**	6	3	2	1	0
**7**	4	1	0	1	0
**8**	6	0	0	0	0
**9**	3	0	0	0	0
**10-11**	2	1	0	0	1
**12-13**	5	2	1	0	1
**14-15**	2	0	0	0	0
**16-17**	0	0	0	0	0
**18-19**	1	1	0	0	1
**20+**	3	0	0	0	0

Table S5. Distribution of the gold standard daily dosage in training and validation sample

Gold standard dosage	Frequency N (%)	
	Training sample	Validation sample
0.5	2 (0.02)	8 (0.9)
1	3347 (41.13)	613 (69.27)
2	1497 (18.4)	148 (16.72)
3	657 (8.07)	52 (5.88)
4	434 (5.33)	22 (2.49)
5	191 (2.35)	9 (1.02)
6	266 (3.27)	6 (0.68)
7	82 (1.01)	4 (0.45)
8	137 (1.68)	6 (0.68)
9	88 (1.08)	3 (0.34)
10-11	95 (1.17)	2 (0.23)
11-12	92 (1.13)	6 (0.68)
14-15	111 (1.36)	2 (0.23)
16-17	52 (0.64)	0 (0)
18-19	33 (0.41)	1 (0.11)
20+	69 (0.85)	3 (0.34)

Figure S1. Predicted versus gold standard daily dosage for predicting prescribed daily dosage (NLP2 model) among informative prescriptions (a) ADHD medications (b) Methylphenidate and (c) Atomoxetine prescriptions.

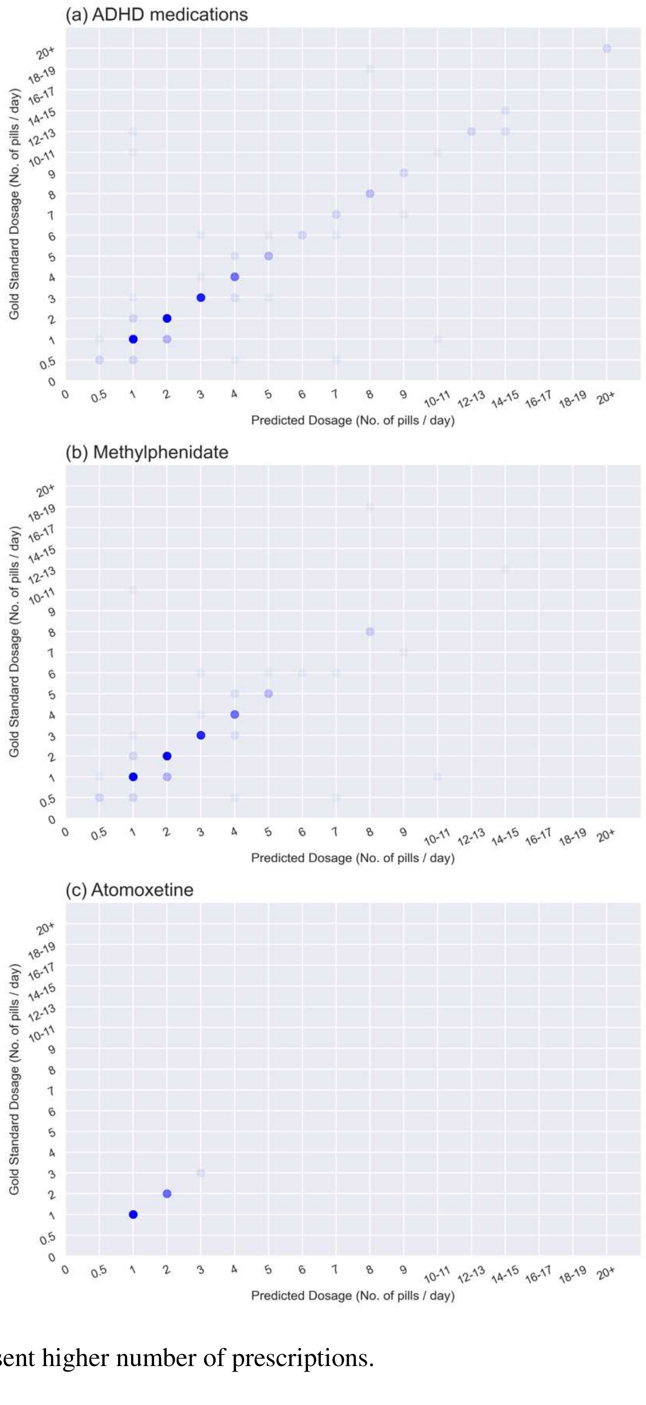

Note: Darker dots present higher number of prescriptions.

Figure S2. Confusion matrix for predicting prescribed daily dosage (NLP2 model) among informative ADHD medication prescriptions.

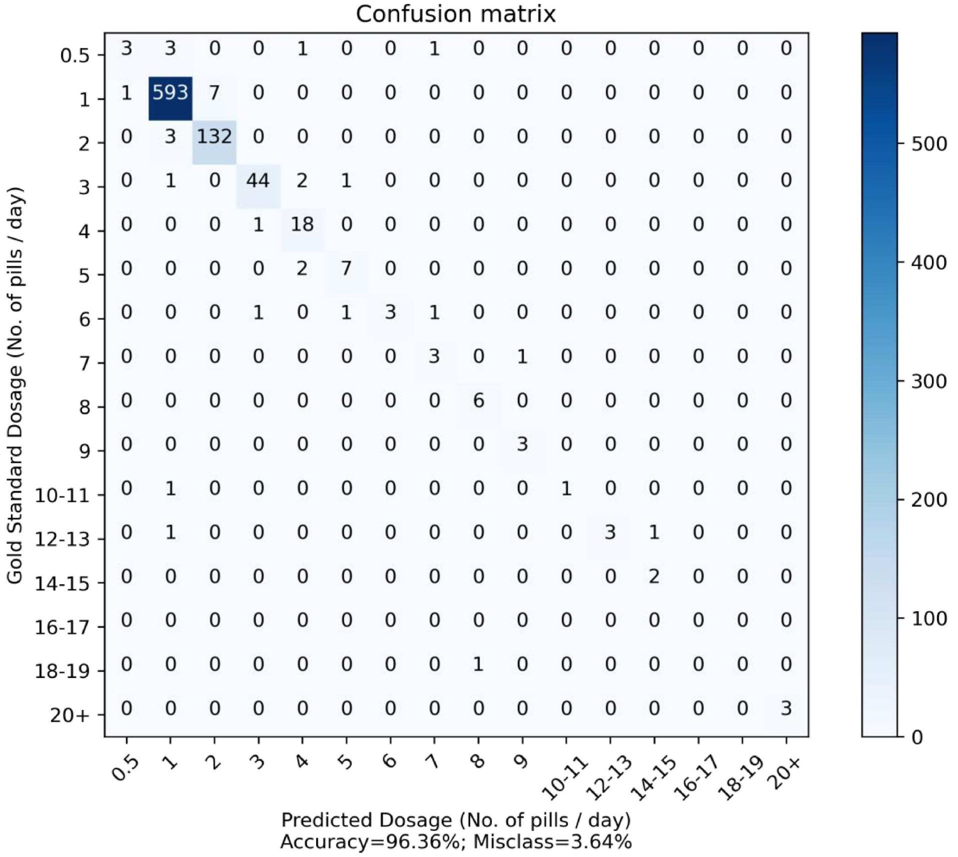

Figure S3. Kaplan-Meier plot of time to treatment discontinuation among incident ADHD medication users in 2013, excluding those with single dispensed prescriptions.

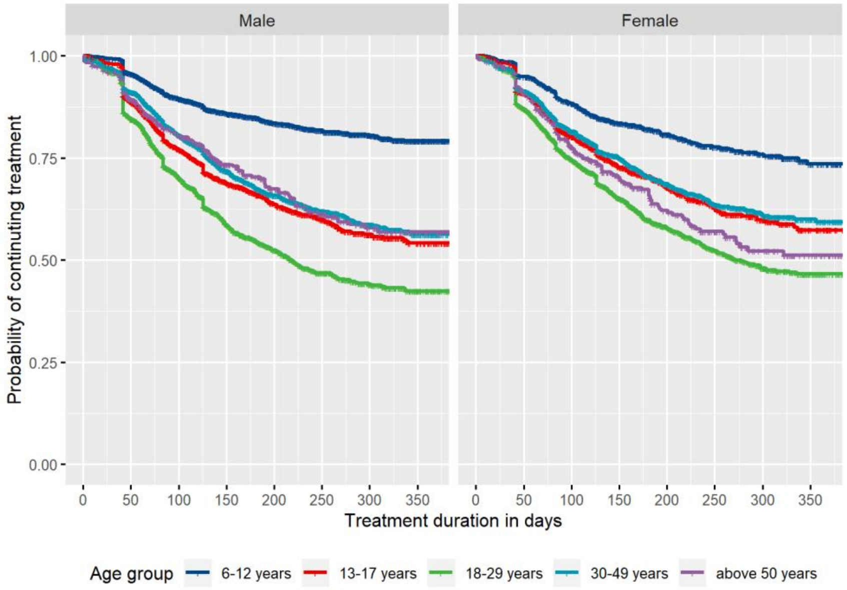

Supplement: Supplementary data [file ebmental-2020-300231supp001.pdf]
